# Sexual Dimorphism in Human Mandibular Canines: A Radiomorphometric Study in South Indian Population

**DOI:** 10.5681/joddd.2011.011

**Published:** 2011-06-14

**Authors:** Rishabh Kapila, K.S. Nagesh, Asha R. Iyengar, Sushma Mehkri

**Affiliations:** ^1^ Senior Lecturer, Department of Oral Medicine and Radiology, Guru Nanak Dev Dental College and Research Institute, Sunam, Punjab, India; ^2^ Principal and Head of Department of Oral Medicine and Radiology, DAPM R.V. Dental College and Hospital, Bangalore, India; ^3^ Professor, Department of Oral Medicine and Radiology, DAPM R.V. Dental College and Hospital, Bangalore, India; ^4^ Lecturer, Department of Oral Medicine and Radiology, DAPM R.V. Dental College and Hospital, Bangalore, India

**Keywords:** Mandibular canine width, radiomorphometric study, sexual dimorphism

## Abstract

**Background and aims:**

The aim of this study was to determine whether variations in the mesiodistal dimensions of mandibular canines had any role in sex determination.

**Materials and methods:**

The study comprised of patients in the 19‒24-year age group (20 males and 20 females). Mesiodistal dimensions of mandibular canines was measured at the maximum mesiodistal width, first intraorally, then on plaster models of the same patient, followed by intraoral periapical radiograph of the same patient. The values were subjected to statistical analysis using t-test.

**Results:**

It might be concluded from the results that there exists a definite statistically significant difference in the mesi-odistal width of mandibular canines when measured for males and females. Moreover, the left mandibular canine showed a greater sexual dimorphism (9.7%) when compared to the right mandibular canine (7.4%).

**Conclusion:**

The present study establishes a statistically significant sexual dimorphism in mandibular canines. It can be concluded that the standard mandibular canine index is a quick and easy method for determining sex and in identification of an unknown individual.

## Introduction

Human teeth are the hardest and chemically the most stable tissues in the body, and are extremely durable even at high temperatures. Teeth can be identified even when the rest of the body has undergone decomposition. They are therefore invaluable for identification on fragmentary adult skeleton. Teeth are readily accessible for examination and since no two teeth have similar morphology, they form an excellent forensic tool for sex determination. The identification of sex is of significance in case of major disasters where bodies are often damaged beyond recognition. Of all the teeth in the human dentition, canines are the least frequently extracted teeth (possibly because of the relatively decreased incidence of caries and periodontal disease).^1^ Mandibular canines are considered to be the key teeth for sexual dimorphism.^2^ Also, canines are reported to withstand extreme conditions and have been recovered from human remains even in air disasters and hurricanes.^1^

Tooth size standards based on odontometric investigations can be used in age and sex determination as human teeth exhibit sexual dimorphism.^3^ Males possess larger tooth crowns than females in contemporary human populations. This may be due to a longer period of amelogenesis for both deciduous and permanent dentitions in males.^4^

Few studies have established that the mesiodistal diameter of lower canine is less in females than males and they have established variations. Hence, the present study aimed to measure the mesiodistal diameter of both mandibular canines so as to establish canine measurement variations in sex determination.

## Materials and Methods

The study comprised of 40 subjects in the 19‒24-year age group, of which 20 were males and 20 females.

The significant exclusion criteria for selection of the study sample were the presence of partially erupted/ectopically erupted teeth, patients with dental/occlusal abnormalities (such as rotation, crowding, occlusal disharmony etc), teeth showing physiologic or pathologic wear and tear (e.g. attrition, abrasion, abfraction, erosion), and patients with deleterious oral habits (like bruxism).

**Figure 1:**
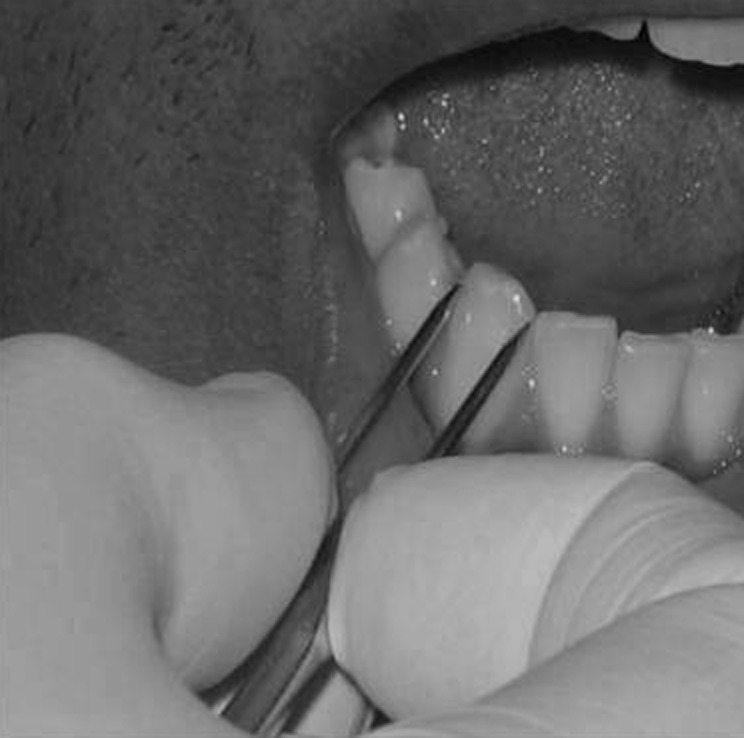
Measurement of mesiodistal width of mandibular canine by clinical examination.

**Figure 2:**
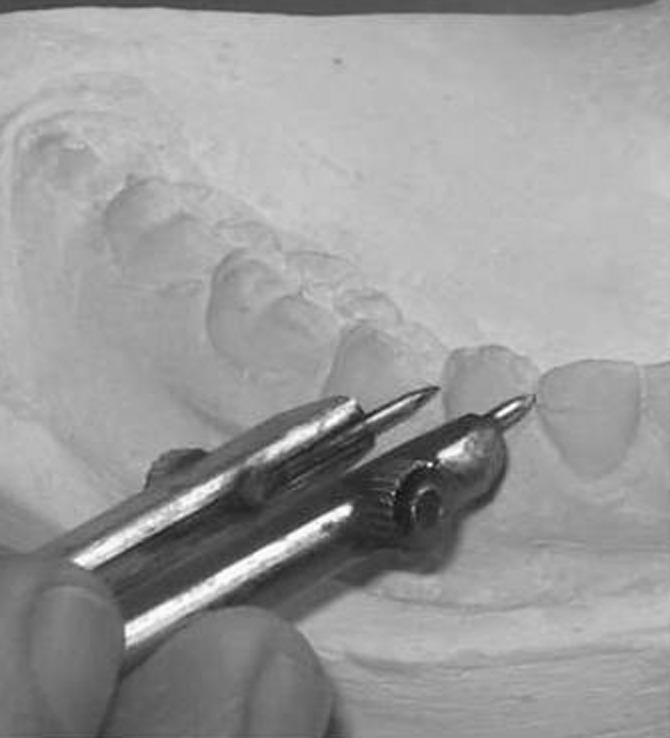
Measurement of mesiodistal width of mandibular canine on plaster models.

**Figure 3:**
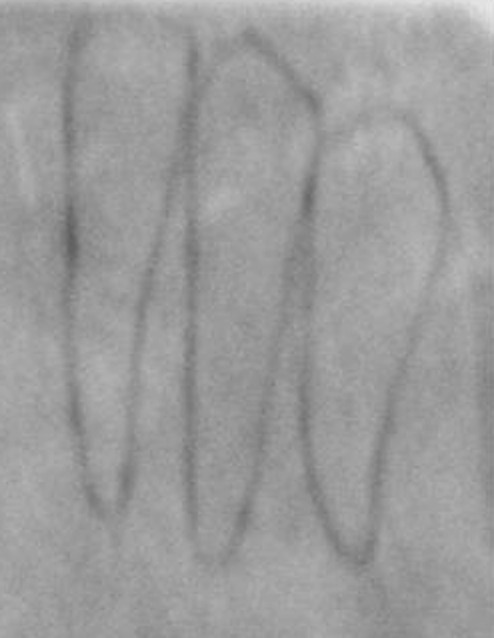
Measurement of mesiodistal width of mandibular canine on tracings of intraoral periapical radiograph.

Mesiodistal diameter of mandibular canines was measured at the maximum mesiodistal width, first intraorally, then on plaster models of the same patient, followed by measurement on tracings of intraoral periapical radiographs (made by paralleling technique for standardization) of the same patient (Figures 1,2 and 3) (Table 1).

**Table 1:** Mean mesiodistal width of mandibular canine obtained by clinical examination, plaster models and by radiographic examination.

Group	Side	Mean value of mesiodistal mandibular canine width (in mm)			P-value
		Clinical Examination	Plaster Models	Radiographic Examination	
Males	Right	7.126	7.04	6.991	<0.001
	Left	7.173	7.133	7.04	
Females	Right	6.626	6.446	6.311	
	Left	6.533	6.46	6.36	

The protocol was reviewed by the institutional ethics committee and the project was undertaken after a signed detailed consent form by the patients.

It was followed by calculation of sexual dimorphism according to the formula given by Garn and Lewis:

**Figure:**
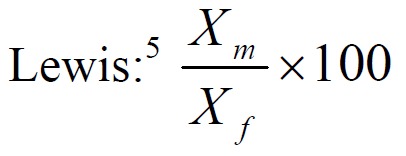


Xm = Mean value of canine width in males

Xf = Mean value of canine width in females

Data was subjected to statistical analysis using ANOVA.

*Statistical Analysis*

The mean values of the mesiodistal width of left and the right mandibular canines in males and females were obtained by clinical examination, measurement on plaster models and radiographic examination and analyzed (Table 1).

The mean value of the mandibular canine width in males and females on the right and left sides were compared using t-test. Moreover, the right and left sides were compared using independent t-test, irrespective of the gender.

The Excel and SPSS (SPSS Inc, Chicago) software packages were used for data entry and analysis.

## Results

From the results of the present study, it is evident that the mesiodistal width of mandibular canines as determined by clinical examination, measurement on plaster models and radiographic examination was statistically insignificant (P > 0.05).

The mean values of mandibular canine widths in males and females on the right and left sides were compared using t-test and were found to be statistically significant (P < 0.0001). The mean value was greater in males as compared to females and left canine was bigger in size than the right irrespective of gender. This was irrespective of whether measurements were made by clinical examination, measurement on plaster models or by radiographic examination.

**Figure 4:**
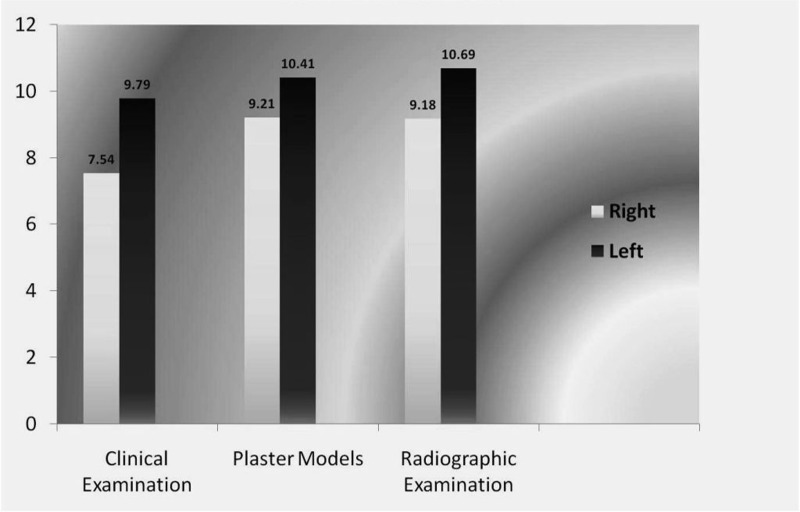
Comparison of sexual dimorphism on the right and left mandibular canines.

The present study also indicates the probability of sex determination to an extent as high as 90% (when the width of either canine was greater than 7 mm, the sex was male). Moreover, the left mandibular canine showed a greater sexual dimorphism (9.7%) when compared to the right mandibular canine (7.4%) (Figure 4).

## Discussion

Mandibular canines are believed to demonstrate the greatest percentage of sexual dimorphism in their mesiodistal width amongst all the teeth.^5-8^

Hashim HA and Murshid ZA in their study on 720 teeth on pretreatment orthodontic casts in a Saudi population aged 13‒20 years noticed that the canines were the only teeth to exhibit dimorphism.^9^ In a continuation of the same study, they also determined that there was no statistically significant difference between the left and right canines, suggesting that measurement of teeth on one side could be truly representative when the corresponding measurement on the other side was unobtainable.^10^ In another study on Saudi Arabian sample of 503 schoolchildren by Al-Rifaiy et al it was reported that the mean values for left and right maxillary and mandibular canine mesiodistal width was less for females than for males, with no statistically significant differences.^11^

Kaushal et al in their study on 60 subjects in a North Indian population found a statistically significant dimorphism in mandibular canines. The mandibular left canine was seen to exhibit greater sexual dimorphism (8.8%) than the right mandibular canine (7.9%). They also concluded that if the width of the canine is greater than 7 mm, the probability of the sex of the person under consideration being male was 100%.^1^ Another study by Nair et al on South Indian subjects concluded that the left mandibular canine with 7.7%, followed by right mandibular canine with 6.2%, shows the maximum sexual dimorphism.^12^ The results of the present study, which was conducted on a South Indian Population, are consistent with the results of above-mentioned studies, where greater sexual dimorphism (9.7%) was exhibited in the left mandibular canine than in the right mandibular canine (7.4%) and when the width of the mandibular canine was greater than 7 mm, the probability of the sex of the person under consideration being male was 90%.

Reverse dimorphism (where the females showed larger teeth than males) was found in studies carried out by Acharya and Mainali^13^ on mandibular second premolar in Nepalese population and by Yuen et al1^4^ on mandibular incisors in a longitudinal study on Chinese population.

The present study establishes a statistically significant sexual dimorphism in mandibular canines. Determination of sex by mesiodistal measurement of mandibular canines is a relatively quick, easy and inexpensive method, and can aid in identifying persons from fragmented jaws and dental remains.

It must be noted, however, that the method of sex determination via canine measurement has its limitations; the sex of the subject to whom the fragment of the mandible belongs can be determined satisfactorily only when the fragment is found in the geographical area where the subject was born.

## Conclusion

The emerging field of forensic odontology in India relies a lot on such inexpensive and easy means of identifying persons, and in such situations the dentist can be called upon to render expertise in forensic science. A database may be established of dental morphometric measurements using intraoral periapical radiographs with a view to determine variations among large populations that may be beneficial for anthropological, genetic, legal, and forensic applications.
